# Medical Sketches Taken in the Royal Navy

**Published:** 1812-02

**Authors:** James Robinson Scott


THE
### Medical and Physical Journal.

2 OF VOL. XXVII.] FEBRUARY, 1812. [NO. 156.

*For the Medical and Physical Journal.*

### MEDICAL SKETCHES *taken in the* ROYAL NAVY, *by* JAMES ROBINSON SCOTT.

### No. I.

(To be continued.)

### *Depletion by the Lancet.*

I HAVE for some time past been impressed with a conviction, that, in the great number of diseases which fall under the observation of practitioners in the navy, their removal, when attended with acute symptoms, and the prevention of subsequent chronic affections, might be more frequently accomplished, if more serious attention was given to the subject of copious evacuation by venesection.

That a mode of practice, founded on rational theory, established by long experience, replete with safety in the hands of the judicious, and the anchor of Hope in many alarming diseases, should be neglected in any case, where the most obvious and pressing symptoms demand it, may be matter of surprize; and most justly when the present improved state of medical science is considered, and the rich stores of information on all subjects connected with it, which are open to every individual professing the healing art.

If the student's observations were more particular and minute, and his mind unbiassed with favorite hypotheses, then might his practice be more successful. The habit of taking general views of diseases, and of treating them too much on general principles, which many practitioners acquire, must fill every reflecting mind with regret, as it is undoubtedly a source of great error.

Theory, however, in this science, is indubitably of the very last importance: many who pride themselves on their rejection of reasoning, and are attentive only to facts, cannot often discover the most palpable causes of disease. Unable to trace remote consequences from insulated facts, they blindly grope or force their way through, to the imminent danger of those in their power, and to their own discredit.

Although numbers of the readers of the Medical and Physical Journal may not find much matter either new or attractive in this sketch, yet, from the facts which decided my intention of laying it before the public, (some of which it will be necessary for me to state,) I hope my motives may not be mistaken. I solicit the indulgence of the enlightened and scientific part of the profession; and, if these hints should be the means of lessening the sufferings of one individual, I shall feel a satisfaction in having ventured to make them known.

Daily do we meet with instances of irreparable injuries produced by excessive arterial action in the human system; how often do we see patients cut off by phthisis, originating in neglected thoracic inflammation—some sinking under the pressure of extravasated blood, from the same cause; and others falling into enteritis, when copious evacuation by the lancet, if timely used, might perhaps have prevented these formidable diseases.

When some important viscus labours under distention of its blood-vessels, with increased action, having its proper functions, as an organ, suspended, or very imperfectly carried on, its powers suffering exhaustion and its structure derangement; we should be prompt in abstracting a proper quantity of that fluid, which, from the morbid state of the part, has become a destructive stimulus to it; to lessen which, is the best and only direct method of giving the moving fibres time to recover their diminished power; and, by moderating their increased action, enable them to free themselves from an injurious and accumulating burthen.

I have often witnessed a strange reluctance to use the lancet, beyond certain limits, which the cases peremptorily demanded to be exceeded. Within these bounds, which the practitioner fancied should not be transgressed, the benefit resulting to the patient from the evacuation could be very small indeed, if any. This dread of depletion, I could, on reflection, attribute to nothing but a habit of prejudice against the use of it, or to a total want of discrimination. In the navy there is presented to the young practitioner an extensive field for observation. Since I entered this service I have had an opportunity of seeing the symptoms, progress, and treatment, of a great many cases of disease to which seamen are subject. With a number of practitioners, I have witnessed the good effects of very copious venesection; and by others, where the cases appeared equally to demand great depletion, I have seen it thrown aside altogether! I have little doubt, however, but that the majority of medical gentlemen in the navy will agree with me, that both reason and experience are on his side, who, in such affections as I am about to mention, uses venesection freely; and, although there occur many instances in which the neglect of this evacuation produces dreadful consequences, I can assert, that, as as far as my observations have extended, I have not seen a case where the practitioner had injured his patient by bleeding; nor have I met in my reading with a decided instance, where one possessed of common talents had done mischief by venesection.

I am well aware that our hospital physicians experience frequent cause of regret, by having cases under their care, where, in the original treatment, venesection has been too long postponed, too feebly enforced, or altogether omitted. Of this I have had ocular proof, and experience the bad effects of this evacuation being neglected, in my own person, when I laboured under thoracic inflammation.

There are many, who, from fear of debilitating the system by large evacuations in the commencement of inflammation, abstract a quantity of blood, much too small to have that effect on the moving powers which the case demands; while the disease continues to gain ground and to proceed to its acmé, notwithstanding these small evacuations are repeated.

There are, undoubtedly, certain states of the constitution attending many acute diseases, which must deter the practitioner from using the lancet freely; these I need not point out: in naval practice they are not numerous, the principal diseases there belonging to Cullen's class pyrexiæ, and particularly to the order phlegmasiæ of that class; in which, evacuations of blood are chiefly important, and in a direct ratio to the promptitude with which they are put in force on the immediate accession of the symptoms. Having premised so far generally, I will take a view of some of those deviations from health which demand very copious evacuations of blood; of the extent to which they may, and often must, be carried to ensure success; and of the great utility in bleeding freely on the accession of the disease, instead of beginning with small quantities, although they may be frequently repeated.

The necessity for considering this part of the subject must be fully obvious, when there are some professional men, who are either not aware of the utility of this kind of evacuation, or are afraid, from certain opinions hereafter to be mentioned, of doing more than what is tantamount to no evacuation, or nearly so; such as in a stout subject, attacked with pneumonia, taking away eight or ten ounces of blood from the arm, when they should take twenty-four or thirty ounces; perhaps repeating that V.S. of eight ounces once in twelve or twenty-four hours, notwithstanding the symptoms continue unabated in violence; and such cases I have witnessed. How often does it happen, in subjects by no means plethoric, that the alarming symptoms at first, and the subsequent rapid progress of the complaint, require from a pound to two pounds of blood to be taken away in the commencement, and perhaps from sixteen to twenty-four ounces daily for many days together, before the symptoms abate in violence? The quantity of blood thus lost, has, in many instances, been truly surprising, the diet being at the same time very low, yet those patients have received no permanent injury from such evacuations. I have read much and heard a great deal said about the injuries done to the system by the loss of so much vital stimulus: but let us consider, that a disease exists whose chief stimulus is the blood circulating in the part affected; that this disease must of itself produce, if it be neglected, dangerous and fatal debility; that it is better weakness arise by depletion, than to let it be caused by protracted arterial action: let us consider that it is voluntary muscular exertion, healthy perspiration, &c. which require chiefly stimulus and nutrition from the circulating mass; and that, when the patient is in a state of rest, with the perspiration perhaps obstructed, both these can in a very great degree be dispensed with, until health is restored.

The regularity of action between the exhalent vessels of the lungs, and surface of the body, is of the greatest importance to the health of the system. In pneumonia, when the occasional cause is exposure to cold, (which is generally the case,) acting on predisposition, there is produced in the extreme vessels a diminished state of action; the effect is obstructed perspiration; the mass of blood in the extreme vessels being very considerable, is determined to the internal and larger vessels. Perspiration from the general surface being suppressed, the lungs have to perform more of the exhalent action than they were accustomed to, and are thrown into unusual efforts. Owing to the determination of blood from the extreme vessels, an increased action with diminished power is produced in the heart and great vessels; the blood accumulated in them is propelled into the most vascular parts; the lungs, being predisposed to inflammation, receive it more rapidly, their vessels are distended, their actions frequent, and their power lessened; pain proves another stimulus. The habit of increased action, thus formed in the system, continues; and caloric is accumulated from the rapidity of the circulation producing so much oxygen, as well as other causes dependent on the nervous system.

I need scarcely remark, from this view of the disease, that to divert the blood from the lungs, and restore the cuticular perspiration, are the two grand indications of cure.

Medicines, acting on the skin through the medium of the stomach, restore propriety of action to its exhalents, diminish that of the heart and great vessels, cool the body by evaporation, determine to the extreme vessels, and thus slowly restore the balance of circulating action in the system.

Purgatives, by determining to the intestines, abstract fluid from the system, and indirectly favour the cure of the complaint.

But, by copious blood-letting, we rapidly accomplish all these ends; we lessen quickly the mass of blood, and proportionably the quantity accumulating in the lungs. Relieved from their burthen, the heart and great vessels have their actions diminished; perspiration becomes regular; sudden ease is the result, even before the lungs have had time to be injured, and we generally find that this simple practice has saved our patient infinite uneasiness. We still use antimonials as auxiliaries, but they should never supersede venesection. When perspiration soon occurs, we should not allow this alone to satisfy us, let it be encouraged: but, although nature often does thus much towards restoring herself, yet, when inflammation has once begun, she must be promptly assisted. When bleeding is used largely, and the sensorium happens to be affected, i. e. if there is giddiness, vomiting, or deliquium animi, the effect on the nerves and stomach may produce a state of the system, inimical to the continuation of the diseased action. This state of the stomach, may produce, by sympathy with the skin, a desired effect on the perspiration.

I by no means wish to intimate that we ought always to produce giddiness, vomiting, and deliquium animi. In some patients the loss of a very small portion of blood, will produce suspension of sense. I have seen the loss of eight ounces do so. But this, I think, often arises from the fear of losing blood acting on the system.

Must we not take very considerable quantities of blood away, if we wish to check the determination to any important viscus?

When I see eight or ten ounces of blood taken from a vein, with the intention of affecting the *general* mass, I cannot conceive the utility of the practice. We should consider the great difference between *venous* and *arterial* blood. A small quantity of the latter, taken from that system of vessels which are in the state of excessive action, and from its containing so much of an important stimulus (oxygen), may produce considerable effect. Eight ounces of arterial blood lost daily, would, in this disease, be more likely to be useful, than twenty ounces of venous blood taken from a part distant from the seat of inflammation. But I have observed, that twenty-four ounces of blood, taken away at once, in a full stream, is more effectual in removing moving inflammation, than eight ounces, under similar circumstances, removed at three different periods; and I think the benefit generally resulting from frequent evacuation of small quantities, is not in proportion to their repetition.

It is often found, that, when a copious evacuation is made early, we have not occasion to repeat it more than once or twice, and sometimes not at all. An intelligent surgeon, of long practical experience, observed to me, that he has generally found this the best method of practice. He tells me, that some years ago, aboard ship, he had a patient who was attacked with symptoms of severe pulmonary inflammation, from whom he took in one day above sixty ounces of blood, having by the first bleeding produced deliquium animi. The result was temporary weakness for some days, but an immediate removal of the disease from this great loss of blood: the patient never afterwards experienced any ill effects. In H. M. ship Endymion, in April 1809, on the coast of Spain, I was called to visit the mate of a merchant vessel, under convoy: he was a very athletic man, and had symptoms of pneumonia strongly marked. I took thirty-six ounces of blood from his arm. After the operation, when I did not expect it, syncope occurred; his pulse, which had been 140, sunk by the loss of blood to 60. I put him to bed, and left directions to be informed daily of his case. I enjoined total cessation from motion with diluents, and gave a laxative. Eight days after this, I saw, by accident, the master of the vessel, who told me that he had been altogether well three days after I had left him.

In this disease we should be particularly warned by the presence of pain, hurried respiration, and oppression of the præcordia. Then, if the pulse be quicker than in health, we should bleed instantly, promptly applying the other auxiliaries. Guided by removal of pain, free breathing, and healthy pulse, we should cease from evacuating. The appearance of the blood will generally assist us, but not always. I have reason to believe, that the effects of copious venesection are by no means so much to be dreaded as many suppose: long continued, it must shake, if not destroy, the foundations of life. But in these dreadful diseases we must bleed, or frequently lose, our patient; and, by so doing in proper time, we avoid the risk of being forced, by fatal necessity, thus to debilitate.

Where a patient is obliged to lose much blood, we cannot be too attentive to the circumstance of rest, slight voluntary exertion is then dangerous, the vital actions alone being as much as the system can bear to perform.

